# Intra- and inter-specific reproductive barriers in the tomato clade

**DOI:** 10.3389/fpls.2023.1326689

**Published:** 2023-12-08

**Authors:** Pauline Moreels, Servane Bigot, Corentin Defalque, Francisco Correa, Juan-Pablo Martinez, Stanley Lutts, Muriel Quinet

**Affiliations:** ^1^ Groupe de Recherche en Physiologie végétale, Earth and Life Institute-Agronomy, Université catholique de Louvain, Louvain-la-Neuve, Belgium; ^2^ Instituto de Investigaciones Agropecuarias (INIA-Rayentué), Rengo, Chile

**Keywords:** tomato, self-incompatibility, *Lycopersicon*, unilateral incompatibility, S-RNase, solanaceae

## Abstract

Tomato (*Solanum lycopersicum* L.) domestication and later introduction into Europe resulted in a genetic bottleneck that reduced genetic variation. Crosses with other wild tomato species from the *Lycopersicon* clade can be used to increase genetic diversity and improve important agronomic traits such as stress tolerance. However, many species in the *Lycopersicon* clade have intraspecific and interspecific incompatibility, such as gametophytic self-incompatibility and unilateral incompatibility. In this review, we provide an overview of the known incompatibility barriers in *Lycopersicon*. We begin by addressing the general mechanisms self-incompatibility, as well as more specific mechanisms in the Rosaceae, Papaveraceae, and Solanaceae. Incompatibility in the *Lycopersicon* clade is discussed, including loss of self-incompatibility, species exhibiting only self-incompatibility and species presenting both self-compatibility and self-incompatibility. We summarize unilateral incompatibility in general and specifically in *Lycopersicon*, with details on the ’self-compatible x self-incompatible’ rule, implications of self-incompatibility in unilateral incompatibility and self-incompatibility-independent pathways of unilateral incompatibility. Finally, we discuss advances in the understanding of compatibility barriers and their implications for tomato breeding.

## 1 Introduction

Tomato (*Solanum lycopersicum* L.) is cultivated worldwide and is the second largest horticultural crop after potato (*Solanum tuberosum* L.) (Fernandes et al., 2018). Both species belong to the Solanaceae family, which also comprises other important crops such as eggplant (*Solanum melongena* L.) and pepper (*Capsicum annuum* L.). In 2021, tomato production reached approximately 189 million tons over 5.17 million ha of cultivated area (FAOSTAT, 2023). The main global tomato producers include China, India, Turkey, the USA, Italy, and Egypt. Tomato yield differs greatly among countries, ranging from 1.47 tons per ha in Somalia to 476 tons per ha in the Netherlands (FAOSTAT, 2023). Tomatoes can be eaten raw or processed into sauces, pastes, soups, juice, or powdered concentrate (Gerszberg et al., 2015).

The tomato was first introduced into Europe and Asia from South America in the 16^th^ century and later to Africa, and gained popularity as a crop during the 19^th^ century (Mazzucato et al., 2010). Domestication and subsequent import of tomato led to a genetic bottleneck in the species. According to Miller and Tanksley (1990), the tomato genome contains less than 5% of the genetic diversity observed in its wild relatives. There are a total of 12 wild tomato species, which, along with *S. lycopersicum*, form the *Lycopersicon* clade (Peralta et al., 2008).

Tomato is sensitive to biotic and abiotic stresses. Since the 1930s, biotic and abiotic stress tolerance genes from wild species have been used to improve tomato stress tolerance (Verlaan et al., 2013; Zhang et al., 2017). At present, introgression of genes from wild relatives remains the most effective method to improve tomato traits through breeding (Fischer et al., 2011; Zsögön et al., 2018; Calafiore et al., 2019; Vitale et al., 2023). One of the most well-studied wild tomato species for introgression is *Solanum pennellii*, due to its resistance to various stresses and its strong capacity to hybridize with *S. lycopersicum* (Brog et al., 2019). Other wild tomato species have also been used similarly. For instance, *Solanum chilense* has been used to introgress resistance to Tomato yellow leaf curl virus (Verlaan et al., 2011; Dhaliwal et al., 2020), *Solanum pimpinellifolium* for salt tolerance and resistance to spider mite and late blight (Salinas et al., 2013; Chen et al., 2014; Rao et al., 2015; Bonarota et al., 2022), *Solanum habrochaites* for insect pest resistance as well as drought tolerance (Frelichowski and Juvik, 2001; Arms et al., 2015) and *Solanum neorickii* for powdery mildew resistance (Bai et al., 2003).

A major obstacle to using wild relatives in tomato breeding is the presence of intra- and interspecific reproductive barriers. Intraspecific barriers, known as self-incompatibility (SI), prevent self-fertilization and maintain genetic diversity in species by promoting outcrossing (Kaothien-Nakayama et al., 2010; Fujii et al., 2016; Broz and Bedinger, 2021; Chakraborty et al., 2023). SI barriers rely on self and non-self-recognition mechanisms between pollen and pistil, followed by inhibition of pollen tube development. Self and non-self-recognition is usually controlled by the *S*-locus, which has multiple S-haplotypes (Takayama and Isogai, 2005). Each S-haplotype bears specific male and female S-determinants, which enable discrimination between self and non-self (Fujii et al., 2016).

Angiosperms have two types of SI: gametophytic self-incompatibility (GSI), observed most notably in Solanaceae, and sporophytic self-incompatibility (SSI), which is present in Brassicaceae (Fujii et al., 2016). Wild tomato species can be self-compatible (as in *Solanum neorickii*), self-incompatible (as in *Solanum chilense*) or both depending on the population (as in *Solanum pennellii*), while *Solanum lycopersicum* is self-compatible (Peralta et al., 2008; Grandillo et al., 2011).

Interspecific barriers limit interspecific crosses in communities with co-flowering plants (Tovar-Méndez et al., 2017). In the *Lycopersicon* clade, interspecific barriers manifest as unilateral incompatibility, whereby pollen from one species is rejected from the pistil of another species, but the opposite cross is accepted. Specifically, it usually follows the SC x SI rule: pollen from self-compatible (SC) species will be rejected on pistils of self-incompatible (SI) species (Baek et al., 2015; Fujii et al., 2016; Tovar-Méndez et al., 2017).

In this review, we synthesize current knowledge about reproductive barriers in the tomato clade. First, we address SI: general GSI mechanisms, and more specific GSI mechanisms in Rosaceae, Papaveraceae and Solanaceae with a focus on the Collaborative Non-Self Recognition Model in Solanaceae. Then, we discuss incompatibility in tomato species, including mechanisms underlying the loss of SI and acquisition of SC. Interspecific barriers and unilateral incompatibility in general and specifically in the *Lycopersicon* clade will be addressed, with details regarding the SI x SC rule, implications of SI in unilateral incompatibility, and SI-independent pathways of unilateral incompatibility. Finally, we discuss advances in our understanding of compatibility barriers, as well as their implications for tomato breeding.

## 2 Self-incompatibility

### 2.1 Gametophytic and sporophytic self-incompatibility

Self-incompatibility (SI) is a mechanism in angiosperms that prevents self-fertilization, thereby maintaining genetic diversity (Takayama and Isogai, 2005). Specifically, SI is defined as the incapacity of a sexually capable hermaphroditic seed-plant to generate zygotes through self-pollination (Nettancourt, 2001). SI is present in roughly 40% of flowering plant species and in at least 100 families (Igic et al., 2008). The SI response depends on pollen–pistil self- or non-self-recognition, followed by inhibition of pollen tube development for self pollen. In most species exhibiting SI, pollen–pistil recognition is controlled by a single, highly polymorphic S-locus. The S-locus contains at least two transcriptional units: the male determinant and the female determinant. There are different S-alleles of the S-locus, and an incompatibility response occurs when both pistil and pollen harbor the same S-allele (Takayama and Isogai, 2005).

**Figure 1:**
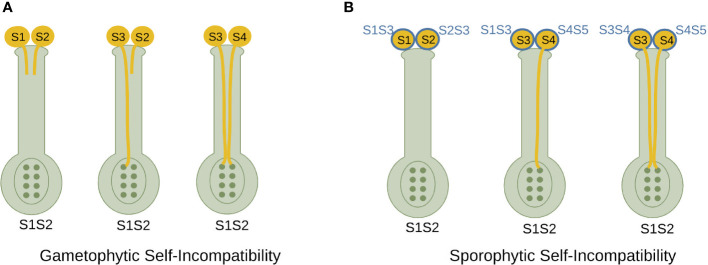
Comparative representation of self-incompatibility mechanisms.**(A)** Gametophytic self-incompatibility. The presence of identical S-alleles in the haploid pollen and diploid pistil leads to the arrest of pollen tube growth, but the presence of different S-alleles leads to fertilization. **(B)** Sporophytic self-incompatibility. Pollen grains bear the S-haplotype products of both parents, which interact with pistil S-haplotype products during the self-compatibility response. In order for fertilization to occur, the pollen S-haplotype must not share either S-allele with the pistil.

Angiosperms have two types of SI: sporophytic SI (SSI) and gametophytic SI (GSI) (**Figure 1**). The two mechanisms differ in the way the pollen SI phenotype is regulated. In GSI, the pollen SI phenotype is determined by its own haploid genome, while in SSI, the pollen SI phenotype is determined by the diploid genomes of the parental donor tissues. This means that in SSI, if the emitting pollen-producing plant has at least one S-allele in common with the receiving plant, all pollen from the emitting plant will be rejected. In contrast, in GSI, pollen will only be rejected if its haploid S-allele is the same as one of the two pistil S-alleles (Takayama and Isogai, 2005; Fujii et al., 2016). In SSI, recognition occurs in the stigma, leading to pollen hydration and germination through the style. Lack of recognition prevents pollen hydration (Fujii et al., 2016; Broz and Bedinger, 2021; Chakraborty et al., 2023; Wang and Filatov, 2023). In Rosaceae, Solanaceae and Plantaginaceae GSI, interaction between male and female SI determinants occurs in the style (Fujii et al., 2016; Broz and Bedinger, 2021; Wang and Filatov, 2023).

## 3 Genetic regulation of self-incompatibility

The genetic control of SI may differ between plant families. The genetic regulation of SSI has mainly been investigated in Brassicaceae, while GSI has mainly been investigated in Papaveraceae, Rosaceae, and Solanaceae.

In Brassicaceae, SSI is controlled by an *S*-locus comprising the female determinant *S-locus protein 11/S-locus Cys-rich* (*SP11*/*SCR*) and the male determinant *S-locus receptor kinase* (*SRK*) (Schopfer, 1999; Takasaki et al., 2000; Fujii et al., 2016; Chakraborty et al., 2023). The *S*-locus is highly linked to *S-locus glycoprotein* (*SLG*), and both are inherited as an S-haplotype (Nasrallah and Wallace, 1967; Nasrallah et al., 1985; Schopfer, 1999; Suzuki et al., 1999; Sehgal and Singh, 2018). The SP11 polypeptide is expressed in the anther tapetum and moves to the pollen coat; SRK localizes to the plasma membrane of stigmatic papilla cells. A specific and direct molecular interaction between SP11/SCR and SRK from the same S-haplotype triggers the incompatibility response. The response occurs in the stigma and causes self-pollen rejection (Conner et al., 1997; Stahl et al., 1998; Schopfer, 1999; Takasaki et al., 1999; Fujii et al., 2016). Thus, in SSI, the incompatibility response is a consequence of self-recognition of S-determinants (Fujii et al., 2016; Broz and Bedinger, 2021). Other factors function downstream of the self-recognition mechanism. *M* locus protein kinase (MLPK) transduces the SI signal, Armadillo repeat-containing 1 (ARC1) ubiquitinates and degrades target molecules, and thioredoxin h-like protein 1 (THL1) and kinase-associated protein phosphatase (KAPP) inhibit SRK to negatively regulate the SI response (Watanabe et al., 1994; Hatakeyama et al., 1998; Cabrillac et al., 2001; Fujii et al., 2016; Muñoz-Sanz et al., 2020; Chakraborty et al., 2023).

Papaveraceae family members exhibit GSI but also use a self-recognition mechanism, which operates at the stigmatic surface (Fujii et al., 2016; Chakraborty et al., 2023). In *Papaver rhoeas*, the *S*-locus female determinant is *Papaver rhoeas stigma S-determinant* (*PrsS*), which encodes a small protein secreted in the stigmatic papilla cells that acts as a signaling ligand (Foote et al., 1994; Wheeler et al., 2009). *Papaver rhoeas* stigma S-determinant interacts with the male determinant *Papaver rhoeas* pollen S-determinant (PrpS), generating a range of physiological responses upon self-interaction, such as Ca^2+^ and K^+^ influx and an increase in cytosolic Ca^2+^ (Wheeler et al., 2009; Wilkins, 2014). These events act on downstream targets and, in turn, lead to programmed cell death (Wilkins, 2014; Chakraborty et al., 2023).

In contrast, the GSI mechanism in the Rosaceae and Solanaceae involves a completely different mechanism, with pollen tube rejection occurring in the style. In S-RNase-based GSI, the *S*-locus contains at least two linked genes. The first gene encodes a glycoprotein with ribonuclease activity (S-RNase), which acts as a female determinant. S-RNase cytotoxic activity causes pollen rejection when the pollen S-haplotype is identical to either of the two S-haplotypes in the pistil. The second *S*-locus gene encodes an F-box protein that acts as the male determinant (Fujii et al., 2016; Broz and Bedinger, 2021; Chakraborty et al., 2023). The name of the F-box protein varies depending on the family: it is called S-locus F-box (SLF) in the Solanaceae and Rosaceae tribe Maleae, and S-haplotype-specific F-box (SFB) in the Rosaceae genus *Prunus*. F-box proteins are best known for their involvement in the Skp, Cullin, F-box-containing (SCF) complex, which recognizes target proteins for ubiquitination and degradation by the 26S proteasome. Along with other findings, this suggests a model in which non-self S-RNases are recognized by the SCF complex and degraded, while self-S-RNases would escape degradation and break down pollen RNA, terminating pollen tube growth (Fujii et al., 2016; Muñoz-Sanz et al., 2020; Broz and Bedinger, 2021; Chakraborty et al., 2023). However, mutations in *Prunus SFB* confer SC, leading to a model for *Prunus* wherein self-SFB protects self-S-RNases from a general inhibitor (Matsumoto et al., 2012).

### 3.1 Gametophytic self-incompatibility in the Solanaceae

Self-incompatibility of Solanaceae family members is under gametophytic control and has mainly been investigated in *Nicotiana*, *Petunia*, and *Solanum* (Fujii et al., 2016; Muñoz-Sanz et al., 2020; Chakraborty et al., 2023). In the Solanaceae family, the *S*-locus contains the *S-RNase* gene encoding the female determinant, along with multiple *SLF* genes forming the male determinant. The number of *SLF* genes varies between species, ranging from 16–20 in SI *Petunia* to 23 in *Solanum pennellii*, and 19 in *Solanum lycopersicum* (Kubo et al., 2015; Li and Chetelat, 2015). Each SLF protein interacts with one or more S-RNases.

**Figure 2:**
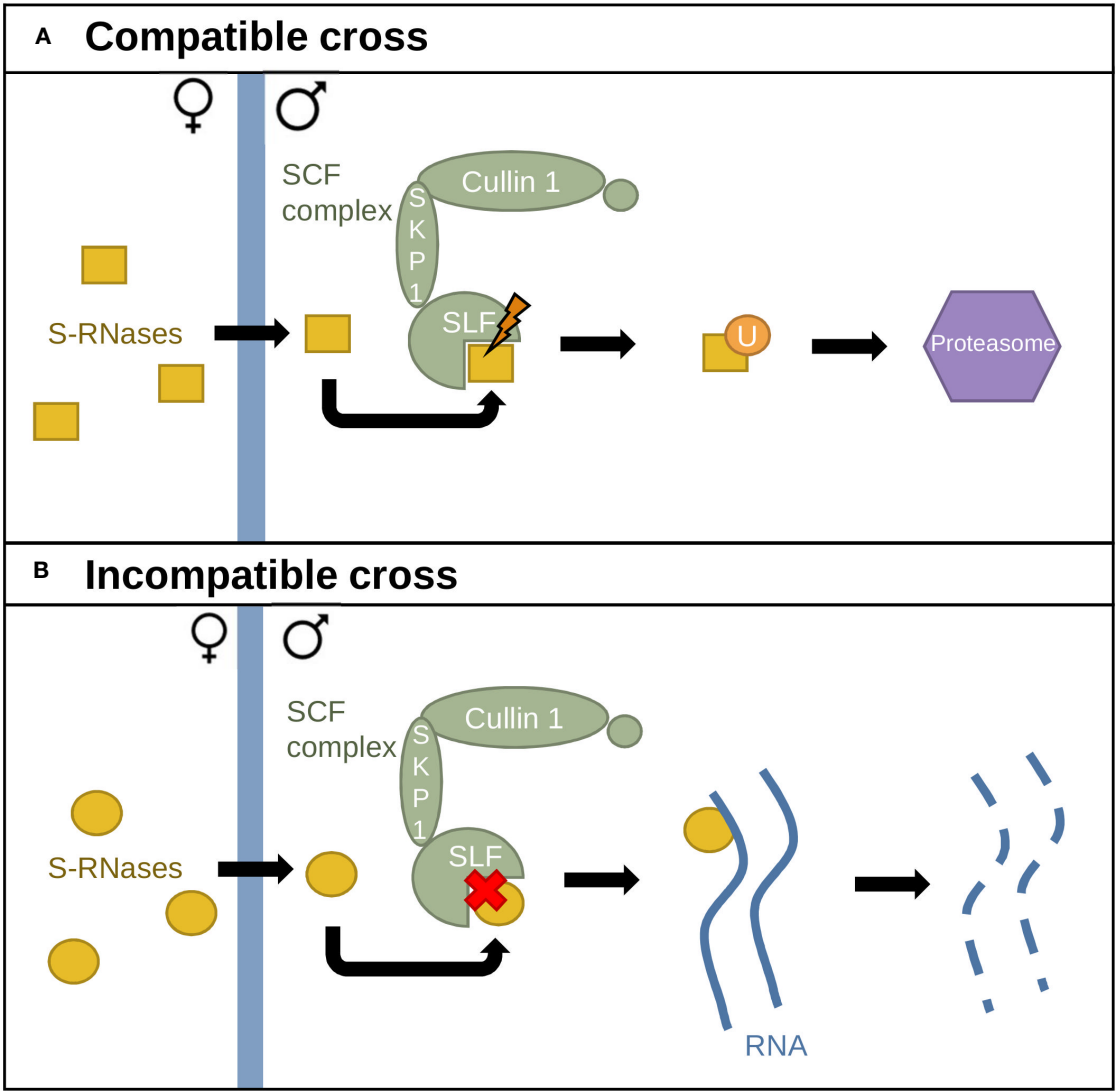
The Collaborative Non-Self Recognition Model. **(A)** Interaction between different S-alleles. S-RNases (yellow) enter the pollen tube and interact with SLF proteins, triggering recognition. S-RNases are then ubiquitinated by the SCF complex and sent to the 26S proteasome for degradation. **(B)** Interaction between identical S-alleles. Lack of recognition between SLF proteins and S-RNases leads to RNA degradation by S-RNases.

Two models have been proposed to explain GSI in Solanaceae: the Collaborative Non-Self Recognition Model (Kubo et al., 2010) and the Compartmentalization Model (Goldraij et al., 2006). In the Collaborative Non-Self Recognition Model, SLF–S-RNase recognition leads to S-RNase ubiquitination and degradation through the 26S proteasome (**Figure 2**) (Fujii et al., 2016). Solanaceae S-RNases possess five highly conserved regions and two hypervariable ones, while the SCF–SLF complex contains a domain fixing the S-RNase hypervariable domain in an S-specific manner, as well as a second domain fixing a conserved region. S-specific fixation leads to S-RNase polyubiquitination by the SCF complex and degradation through the 26S proteasome (**Figure 2A**). In contrast, lack of recognition leads to inhibition of pollen tube growth via S-RNase cytotoxic activity (**Figure 2B**) (Takayama and Isogai, 2005; Kubo et al., 2010; Fujii et al., 2016).

Recent studies have highlighted other factors involved in Solanaceae GSI that are independent of the *S*-locus, known as modifier genes. McClure et al. (1999) described the first modifier gene in GSI: *High Top-Band* (*HT-B*), encoding a small asparagine-rich protein involved in *Nicotiana* S-specific pollen rejection. Two *HT* genes have been identified in *Solanum* (*HT-A* and *HT-B*), although *Solanum lycopersicum* lacks functional HT proteins (Kondo et al., 2002b). The specific roles of each HT protein remain unknown. HT-B is essential for pollen rejection in *Nicotiana*, *Petunia*, and *Solanum*. However, several studies focusing on the *Lycopersicon* clade species revealed a possible overlap between HT-A and HT-B function in SI (McClure et al., 1999; Kondo et al., 2002b; O’Brien et al., 2002; Puerta et al., 2009; Covey et al., 2010; Tovar-Méndez et al., 2014). In *Nicotiana alata*, the protease inhibitor Stigma-Expressed Protein (NaStEP) participates in the SI response by protecting HT-B from degradation in the pollen tube (Jiménez-Durán et al., 2013). The exact role of NaStEP in SI has not yet been determined, but it may inhibit a subtilisin-like component (*NaSubt)* that would otherwise target HT-B during compatible crosses (Cruz-Zamora et al., 2020). A third, pollen-derived protein potentially involved in this mechanism is Self-Incompatibility Pollen Protein (NaSIPP). In incompatible crosses, interaction between NaSIPP and NaStEP causes the opening of a permeability transition pore in the mitochondrial membrane, triggering an energy crisis and interruption of pollen tube growth (García-Valencia et al., 2017). Another factor in *N. alata* that participates in the SI response is a 120-kDa glycoprotein (120K) belonging to the arabinogalactan protein group. Loss of 120K leads to pollen rejection failure (Nathan Hancock et al., 2005). Moreover, a recently identified modifier gene in *N. alata* is *thioredoxin type h* (*NaTrxh*). The *NaTrxh* gene product specifically reduces a highly conserved S-RNase disulfide bond following the S-RNase–SLF interaction in incompatible crosses. This disulfide bond reduction significantly increases S-RNase ribonuclease activity, enabling pollen tube growth arrest (Torres-Rodríguez et al., 2020). How these modifier genes specifically integrate the Collaborative Non-Self Recognition Model has yet to be uncovered.

**Figure 3:**
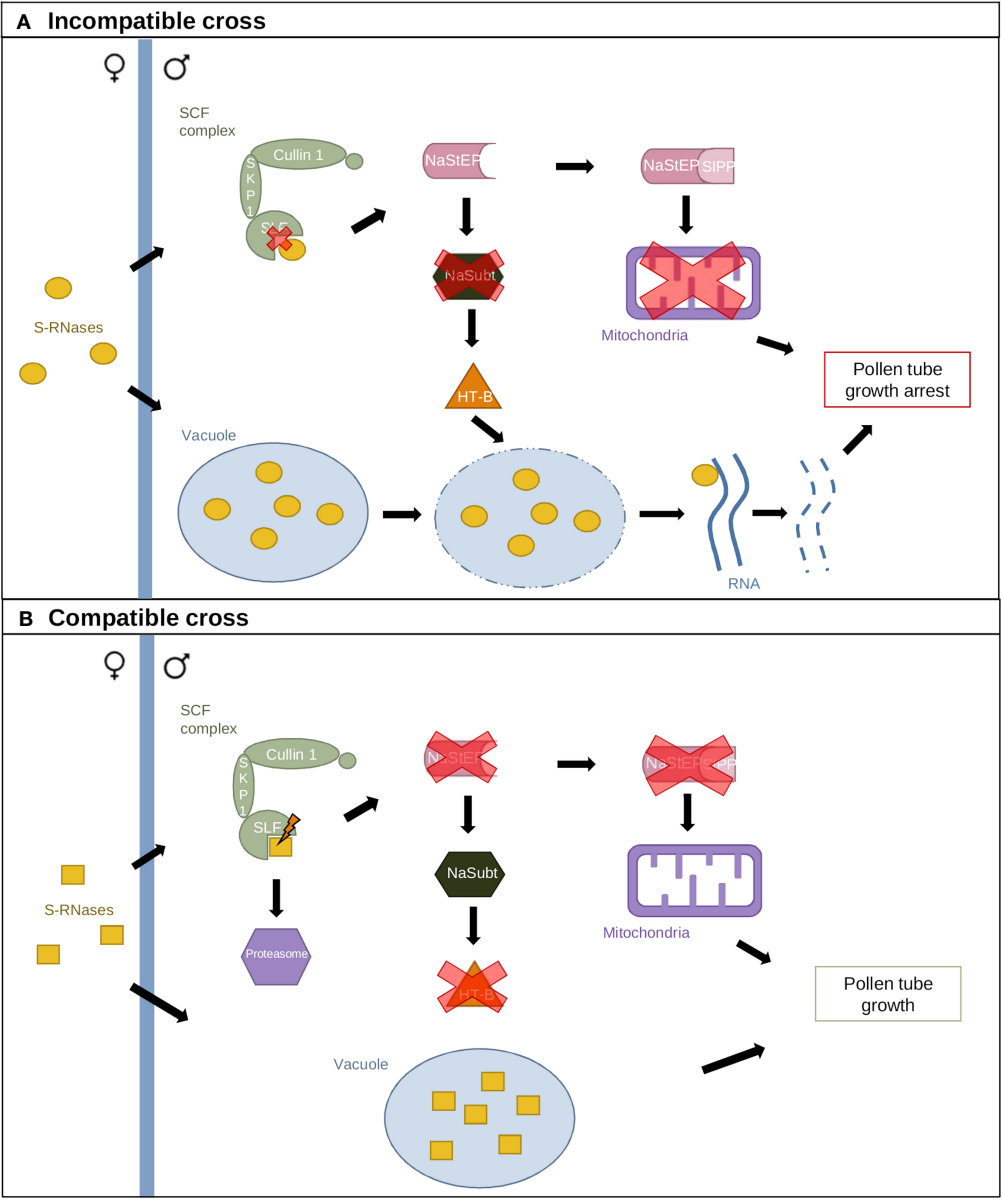
The Compartmentalization Model. **(A)** Incompatible cross resulting from identical S-allele interaction. S-RNases are sequestered in a vacuole, while a small portion interacts with the SCF complex. Lack of recognition between SLF and S-RNases leads to NaStEP maintenance, which inhibits a subtilisin-like component (NaSubt) and maintains HT-B. Interaction between the vacuole and HT-B leads to vacuolar membrane disruption and S-RNase release. NaStEP also interacts with SIPP, destabilizing mitochondria. **(B)** Compatible cross resulting from the interaction between different S-alleles. Recognition between SLF and S-RNases inhibits NaStEP, allowing NaSubt-mediated repression of HT-B. The vacuole and mitochondria remain intact, and pollen tube growth is maintained.

A second GSI model has been proposed in *N. alata*, which includes several modifier genes. The Compartmentalization Model (**Figure 3**) suggests that S-RNases are compartmentalized in vacuoles when they enter pollen tubes to contain their cytotoxic activity (Goldraij et al., 2006). A small portion of S-RNases escape this compartmentalization and interact with the SCF–SLF complex in the cytoplasm, generating the compatibility response (McClure et al., 2011). In incompatible crosses, S-specific interaction leads to NaStEP stabilization, which protects HT-B from degradation (**Figure 3A**). HT-B destabilizes the vacuolar membrane, releasing S-RNases. Furthermore, NaStEP–NaSIPP interaction destabilizes mitochondria. Collectively, these events arrest pollen tube growth. In compatible crosses, S-specific recognition leads to NaStEP inhibition, allowing a subtilisin-like protease to degrade HT-B (**Figure 3B**). HT-B loss results in intact vacuoles and S-RNase sequestration (Goldraij et al., 2006; McClure et al., 2011; Cruz-Zamora et al., 2020).

Later studies in *Petunia hybrida* showed that SLF and S-RNases interact in the cytosol, that S-RNases are polyubiquitinated and degraded by the proteasome in compatible crosses, and that the action of non-self SLF from the SCF complex mediates S-RNase degradation (Liu et al., 2014; Williams et al., 2015). Taken together, these findings support the Collaborative Non-Self Recognition Model. However, except for HT-B, no modifier genes present in *Nicotiana* have been detected in *Petunia*. Thus, compatibility response mechanisms might differ between *Nicotiana* and *Petunia* (Liu et al., 2014; Chakraborty et al., 2023). Still, the two models are not mutually exclusive; a small portion of S-RNases could be compartmentalized, whereas the majority could be degraded in the cytosol (McClure et al., 2011; Liu et al., 2014). Nevertheless, the specific roles of certain modifier genes, such as 120K, remain unknown and the incompatibility mechanisms in GSI need further exploration.

The Collaborative Non-Self Recognition Model is the most widely accepted model in *Solanum* (Li and Chetelat, 2015; Qin et al., 2018; Qin and Chetelat, 2021). Still, compared to *Nicotiana* and *Petunia*, very few studies have focused on *Solanum* SI mechanisms specifically. Additional evidence is needed to elucidate the mechanisms at play in this genus.

### 3.2 Self-compatible and self-incompatible species in the *Lycopersicon* clade

Cultivated tomato (*Solanum lycopersicum*) and its twelve closely related species are grouped in the so-called ‘tomato’ or ‘*Lycopersicon’* clade. This clade is divided into four sub-groups (**Table 1**). The first group (Esculentum) contains *S. galapagense*, *S. cheesmaniae*, *S. lycopersicum* and *S. pimpinellifolium*. The second group (Arcanum) consists of *S. neorickii*, *S. arcanum* and *S. chmielewskii*. The third group (Peruvianum) comprises *S. huaylasense*, *S. peruvianum*, *S. corneliomurelli* and *S. chilense*. The last group (Hirsutum) contains *S. habrochaites* and *S. pennellii* (Pease et al., 2016). All Esculentum group species are self-compatible. In contrast, all species in the Peruvianum group are self-incompatible except for *S. peruvianum*, which includes several facultative SC populations. The other two groups comprise species that are either SI or facultatively SC (Peralta et al., 2008; Grandillo et al., 2011).

**Table 1:** Groups and species in the *Lycopersicon* clade, showing their compatibility relation and S-RNase, HT-A and HT-B activities.

Group	Species	Accession	Compatibility relation	S-RNase	HT-A	HT-B
Esculentum	*S. lycopersicum*		SC	_	_	_
	*S. pimpinellifolium*		SC	_	_	_
	*S. galapagense*		SC	_	_	_
	*S. cheesmaniae*		SC	_	_	_
Arcanum	*S. neorickii*		SC	_	OK	_
	*S. arcanum*		SI/SC			
		LA2157	SC	_	OK	OK
	*S. chmielewskii*		SC	_	OK	_
Peruvianum	*S. huaylasense*		SI	OK	OK	OK
	*S. peruvianum*		SI/SC			
		LA4125	SC	?	?	?
		LA2157	SC	_	?	?
	*S. corneliomulleri*		SI	OK	OK	OK
	*S. chilense*		SI	OK	OK	OK
Hirsutum	*S. habrochaites*		SI/SC			
		LA1777	SI	OK	OK	_
		LA1223	SC	_	_	_
		LA2314	SC	_	OK	_
		LA0407	SC	_	OK	_
	*S. pennellii*		SI/SC			
		LA0716	SC	_	OK	OK

SC, self-compatible; SI, self-incompatible; _, no activity; OK, activity,?, unknown activity.

The SI-to-SC transition can be triggered by several factors that result in loss of SI or gain of SC (Zhao et al., 2022). In the Arcanum group, *S. chmielewskii* is facultatively SC, *S. neorickii* is autogamous and *S. arcanum* is SI except for one autogamous accession, LA2157 (Grandillo et al., 2011; Markova et al., 2017). Both *S. chmielewskii* and *S. arcanum* accession LA2157 have lost pistil S-RNase activity, while *S. neorickii* has acquired a gain-of-function mutation in pollen *SLF* that allows self-recognition (Markova et al., 2017). The Esculentum clade also lost S-RNase activity, and does not possess functional *HT* genes; *HT-A* encodes a truncated peptide, while *HT-B* is not transcribed (Kondo et al., 2002a).

### 3.3 S-RNases in the *Lycopersicon* clade: representation and phylogeny

**Figure 4:**
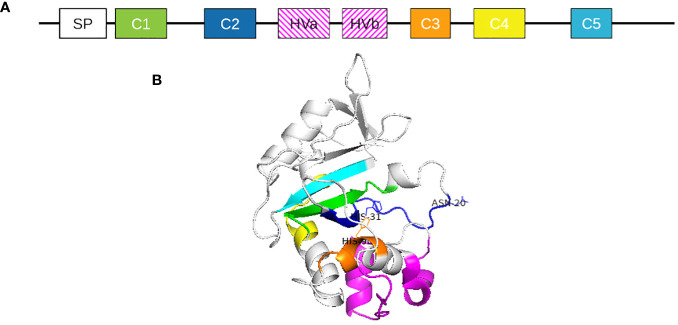
**(A)** Solanaceae S-RNase protein primary structure representation. SP represents the signal peptide. C1 to C5 represent highly conserved regions of the proteins and HVa and HVb represent the two hypervariable regions. **(B)** Three-dimensional structure of S. chilense S2 S-RNase. Conserved regions are indicated in color: green = C1, dark blue = C2, orange = C3, yellow = C4, cyan = C5.

As the *S*-locus in Solanaceae members contains one female determinant but multiple male determinants, S-haplotype characterization is mainly based on S-RNase description (Igic et al., 2007; Kubo et al., 2010; Williams et al., 2015; Broz et al., 2021). S-RNases are 30-kDa glycoproteins secreted by the style and taken up by pollen tubes during their growth (Luu et al., 2000). Solanaceae S-RNases are part of the T2 RNase family: their sequences contain a signal peptide, five conserved regions and two hypervariable regions (**Figure 4**) (Silva and Goring, 2001). S-specificity could be partially attributed to the hypervariable regions. However, the precise role of sequence variation in the hypervariable regions, as well as the specific molecular interactions they mediate, remain unknown (Ioerger et al., 1991; Brisolara-Corrêa et al., 2015).

**Figure 5:**
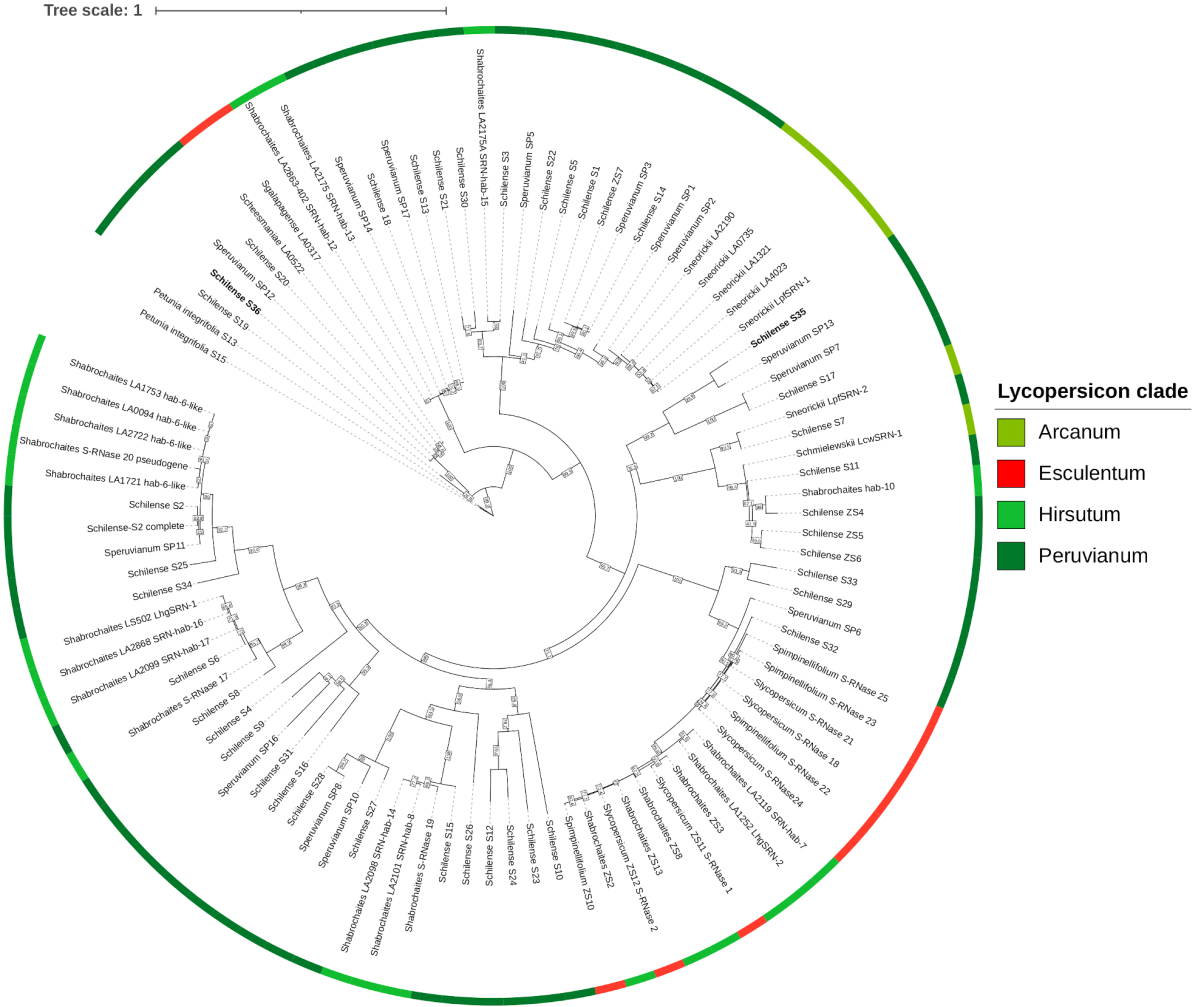
Maximum likelihood phylogram of S-alleles across the *Lycopersicon* clade. Sequences are colored by the group they belong to (See **Table 1** for groups information) and color of their fruits (either green or red, see **Figure 6**). The NCBI accessions of the genes are detailed in **Supplementary Data**.

S-allele diversity in the *Lycopersicon* clade is estimated to range from 10 to 50 S-haplotypes per species, with roughly 35 S-alleles in *S. chilense* (Igic et al., 2007). Identical S-alleles from different species tend to be more closely related to each other than to S-alleles of the same species, as S-allele diversification predates speciation within the clade (**Figure 5**) (Brisolara-Corrêa et al., 2015). Three different scenarios may explain SC acquisition resulting from S-RNase mutation within the clade. The first involves direct gene-disrupting mutations, such as gene deletion, frameshift mutations or nonsense mutations, that generate non-functional *S-RNase* genes. The second scenario involves expressed S-alleles that are translated into proteins that harbor substitutions in crucial amino acids, rendering them non-functional. The third scenario involves S-alleles that have been transcriptionally silenced (Broz et al., 2021).

## 4 Unilateral incompatibility

### 4.1 Interspecific reproductive barriers and unilateral incompatibility

Hybridization amongst individuals from different species can lead to poorly adapted or nonviable offspring due to genetic incompatibilities arising from species divergence (Lewis and Crowe, 1958; Nettancourt, 2001; Wang and Filatov, 2023). Such interspecific reproductive barriers (IRBs) serve to limit outbreeding and can be either passive mechanisms such as differences in matching of genetic systems leading to a lack of fit between partners, referred to as incongruity, or active mechanisms such as pollen rejection, referred to as incompatibility (Lewis and Crowe, 1958; Hogenboom et al., 1997; Nettancourt, 2001; Broz and Bedinger, 2021).

Because IRBs function to prevent self-fertilization, some SI barriers play roles in interspecific incompatibility (Li and Chetelat, 2010; Broz and Bedinger, 2021; Wang and Filatov, 2023). Accordingly, SI species exhibit a stronger inclination to actively reject interspecific pollen than SC species (Lewis and Crowe, 1958). This phenomenon has been described as unilateral incompatibility (UI) (Lewis and Crowe, 1958; Nettancourt, 2001). Unilateral incompatibility is a type of interspecific incompatibility in which pollen from one species is rejected by another species’ pistil, while the opposite cross is fertile (Nettancourt, 2001). Unilateral incompatibility often follows the SI x SC rule, whereby pollen from an SI species is accepted by the pistil of an SC species, while the opposite cross is rejected (Lewis and Crowe, 1958). This type of barrier has been described in families such as the Brassicaceae and Solanaceae (Lewis and Crowe, 1958; Hiscock and Dickinson, 1993; Broz and Bedinger, 2021).

### 4.2 Unilateral incompatibility in the *Lycopersicon* clade

**Figure 6:**
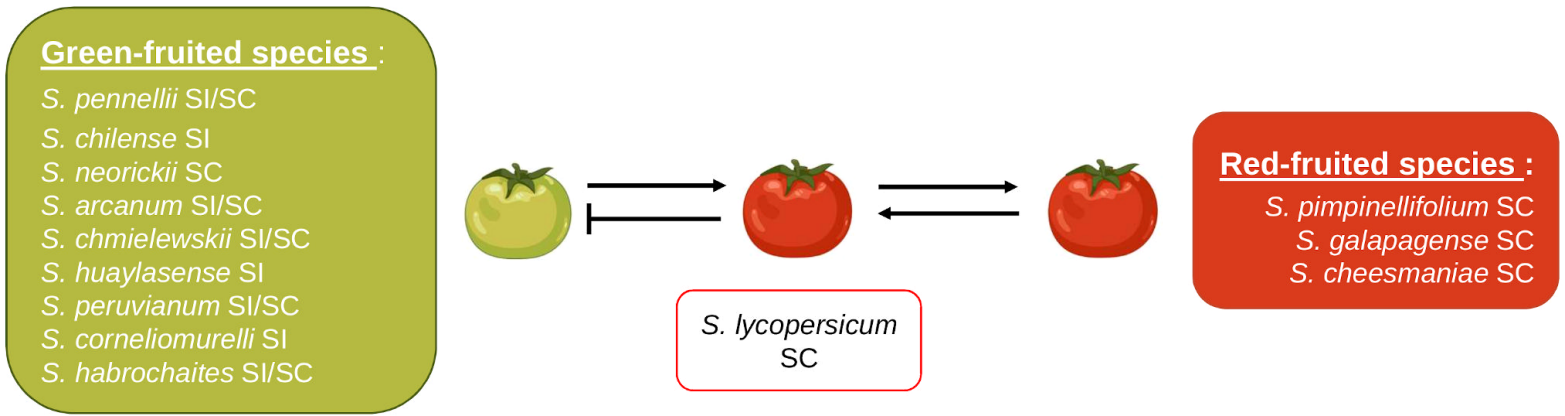
Interspecific compatibility of *S. lycopersicum* with other members of the *Lycopersicon* clade. The blunted arrow represents an incompatible cross and regular arrows represent compatible crosses.

Interspecific reproductive barriers have been observed within the *Lycopersicon* clade, but they may differ among species. Unilateral incompatibility has been particularly investigated in tomato species (Li and Chetelat, 2010; Tovar-Méndez et al., 2014; Tovar-Méndez et al., 2017; Qin et al., 2018; Muñoz-Sanz et al., 2021; Qin and Chetelat, 2021). In the tomato clade, the SI x SC rule often manifests as red-fruited species exhibiting SC and green-fruited species exhibiting SI (**Figure 6**); therefore, red-fruited species may be used as the female parent in crosses with green-fruited species, but the reverse is not true. All red-fruited species belong to the Esculentum group and lack functional S-RNases and HT proteins that prevent interspecific crosses (Grandillo et al., 2011; Tovar-Méndez et al., 2014; Baek et al., 2015). Furthermore, the tomato clade is particularly useful in studying UI since its subgroups show different levels of IRBs. In addition to the Esculentum group showing hardly any IRBs, the Arcanum group displays fewer IRBs than the Peruvianum and Hirsutum groups (Bedinger et al., 2011; Muñoz-Sanz et al., 2021).

Studies involving members of the tomato clade have shed light on SI-dependent and SI-independent mechanisms of UI. UI in tomato species has been most studied in *S. pennellii*. This species includes SI accessions as well as an accession with very low S-RNase levels, two characteristics that enable the study of S-RNase-independent UI pathways (Tovar-Méndez et al., 2014; Tovar-Méndez et al., 2017; Qin et al., 2018; Muñoz-Sanz et al., 2021; Qin and Chetelat, 2021).

Tovar-Méndez et al. (2014) showed that expression of *S-RNase* with either *HT-A* or *HT-B* could restore IRBs in *S. lycopersicum* when crossed with other red-fruited species.

In addition to S-RNases and HT proteins, other factors involved in SI also affect the UI response. Pollen-wise, a single *SLF* transgene, *SLF-23*, in combination with functional *Cullin1*, has been shown to be responsible for pollen recognition and rejection in *Solanum* sect. *Lycopersicon* UI. This finding is in agreement with the SI x SC rule, as all green-fruited species express both functional *SLF-23* and *Cullin1*, while no red-fruited species possess both functioning genes (Li and Chetelat, 2010; Li and Chetelat, 2015).

SI-independent UI pathways have also been recently described. Indeed, *S. pennellii* accession LA0716 and *S. habrochaites* accession LA1927 show very low S-RNase activity, but still reject *S. lycopersicum* pollen. Furthermore, a nonsense mutation was detected in the *S. habrochaites HT-B* gene, whereas the *HT-A* gene product was functional (Covey et al., 2010). *HT* suppression in *S. pennellii* LA0716 enabled deeper penetration of *S. lycopersicum* pollen tubes into the style, and *HT* suppression in *S. habrochaites* LA0407 and *S. arcanum* LA2157 (two S-RNase-deficient accessions) allowed ovary penetration and hybrid production (Tovar-Méndez et al., 2017). Thus, HT proteins may be involved in S-RNase-dependent and S-RNase-independent UI pathways (**Table 2**).

**Table 2:** Genes potentially involved in unilateral incompatibility in the *Lycopersicon* clade.

Gene	Abbreviation	S-RNase dependent?	Pollen- or pistil-side?	Literature
*High Top A* & *High Top B*	*HT-A* & *HT-B*	Yes and No	Pistil	(Covey et al., 2010; Tovar-Méndez et al., 2017)
*Farnesyl Phosphate Synthase 2*	*FPS2*	No	Pollen	(Qin et al., 2018)
*Ornithine Decarboxylase 2*	*ODC2*	No	Pistil	(Qin and Chetelat, 2021)
*Defective in Induced Resistance 1-like*	*DIR1L*	No	Pistil	(Muñoz-Sanz et al., 2021)
*Kunitz-type Protease inhibitor*	/	Unknown	Pistil	(Broz et al., 2017)
*Putative Arabinogalactan Protein*	/	Unknown	Pollen	(Broz et al., 2017)

Other factors have also been linked to the S-RNase-independent way (**Table 2**). In *S. pennellii* LA0716, the pollen-derived protein farnesyl phosphate synthase 2 (FPS2) was shown to be involved in UI pollen rejection, with *FPS2* expression being 18-fold higher in *S. pennellii* LA0716 than in *S. lycopersicum* (Qin et al., 2018). Ornithine decarboxylase 2 (ODC2), an enzyme catalyzing the conversion of ornithine into putrescine in polyamine biosynthesis, was later identified as the pistil-derived factor that interacts with FPS2. There are four copies of *ODC2* in *S. pennellii* but only one in *S. lycopersicum*. Furthermore, *ODC2* genetically interacts with *HT* genes to strengthen pollen rejection (Qin and Chetelat, 2021). Another S-RNase-independent pistil-derived UI factor, *Defective in Induced Resistance 1-Like* (*DIR1L*), contributes to *S. lycopersicum* pollen rejection in *S. pennelli* LA0716. A deletion in the *DIR1L* coding region was identified in the Esculentum and Arcanum groups (Muñoz-Sanz et al., 2021). Transcriptomics analyses of *S. habrochaites* also highlighted other factors potentially involved in UI, such as a Kunitz-type protease inhibitor and a putative pollen arabinogalactan protein (Broz et al., 2017).

Mechanisms of UI are multiple, resulting from various pollen–pistil interactions. A complete picture of this interspecific barrier remains to be uncovered (Tovar-Méndez et al., 2017; Qin et al., 2018; Muñoz-Sanz et al., 2021; Qin and Chetelat, 2021).

## 5 Discussion

Understanding reproductive barriers in plants sheds light on the establishment of reproductive isolation, which is a crucial aspect of speciation (Bedinger et al., 2011). Moreover, reproductive barriers limit the use of wild relatives for crop improvement. Accordingly, overcoming such barriers would facilitate the use of wild relative germplasm in plant breeding (Bedinger et al., 2011; Muñoz-Sanz et al., 2020).

Several types of incompatibility barriers are seen in the *Lycopersicon* clade. On the one hand, gametophytic self-incompatibility prevents inbreeding on an intraspecific level, and on the other hand, unilateral incompatibility limits outbreeding. Important discoveries have expanded our comprehension of the mechanisms underlying both barrier types, especially in *Lycopersicon* clade UI. However, GSI has mainly been investigated in *Nicotiana* and *Petunia* (Solanaceae). Since GSI also plays a role in UI, additional studies in *Solanum* are needed to determine how SI mechanisms unfold in this genus, and by extension, in the *Lycopersicon* clade.

The tomato clade is an excellent model to study reproductive barriers (Bedinger et al., 2011). It comprises species with different compatibility barriers, facilitating the study of both SI and UI. Furthermore, *Solanum lycopersicum*, one of the most important agricultural crops worldwide, could directly benefit from advances in overcoming reproductive barriers to introgress genes of interest from wild relatives (Bedinger et al., 2011; Muñoz-Sanz et al., 2020). Moreover, insights on reproductive barriers from the *Solanum* model could be extended to other members of the Solanaceae family, as well as more distant genera. It has recently been suggested that *Prunus* could present linkages in SI and UI. (Morimoto et al., 2019). Thus, the analysis of reproductive barriers in *Prunus* could directly benefit from progress made in the *Lycopersicon* clade (Bedinger et al., 2011; Morimoto et al., 2019; Muñoz-Sanz et al., 2020).

## 6 Conclusion

In the last two decades, major advances have expanded our knowledge of incompatibility barriers in the *Lycopersicon* clade. Models explaining self-incompatibility mechanisms have been proposed, such as the Collaborative Non-Self Recognition Model and the Compartmentalization Model in S-RNase-based GSI. Factors participating in these SI mechanisms have been uncovered, such as HT, NaStep, NaSIPP, 120K and NaTrxh. Nevertheless, the detailed mechanisms underlying self-incompatibility and the clear roles of these factors have yet to be unraveled. So far, no model explaining unilateral incompatibility in the *Lycopersicon* clade has been proposed, although multiple factors and UI pathways have been identified. Much remains to be learned about this interspecific barrier, in the hope of using the complete array of wild tomato species for tomato breeding.

## Author contributions

PM: Conceptualization, Investigation, Methodology, Visualization, Writing – original draft, Writing – review & editing. SB: Conceptualization, Supervision, Visualization, Writing – review & editing. CD: Investigation, Visualization, Writing – review & editing. FC: Investigation, Software, Visualization, Writing – review & editing. JM: Funding acquisition, Project administration, Resources, Writing – review & editing. SL: Funding acquisition, Project administration, Supervision, Writing – review & editing. MQ: Conceptualization, Funding acquisition, Project administration, Supervision, Writing – original draft, Writing – review & editing.

## Supplementary material

Supplementary Table 1 list of the genebank accessions of the S-RNAses genes presented in .
